# Augmenting the band gap of iron diselenide pyrite via ruthenium alloy integration

**DOI:** 10.1016/j.heliyon.2024.e25965

**Published:** 2024-02-14

**Authors:** Eman A. Alghamdi, Refka Sai

**Affiliations:** Department of Physics and Astronomy, King Saud University, Riyadh, 11451, Saudi Arabia

**Keywords:** Thin films, Photovoltaic, Electrical, XRD, Optical, Ruthenium alloying$\text{Fe}{\text{Se}}_{2}$

## Abstract

The study aimed to enhance the properties of thin $Fe{Se}_{2}$ films by incorporating ruthenium through spray pyrolysis. Films were deposited on pre-heated glass substrates and subjected to controlled heating in a selenium-rich environment. X-ray diffraction analysis confirmed the presence of $Fe{Se}_{2}$ phase. Films with specific ruthenium ratios showed notable improvements in optical attributes, including increased absorption coefficient and a higher direct band gap, aligning with desired values for photovoltaic applications. Hall Effect measurements revealed N-type conductivity with varying concentrations and temperature-dependent electrical properties. The results highlight the efficacy of ruthenium as a promising alloying candidate for developing photovoltaic materials, emphasizing the versatility of the produced films across multiple domains.

## 1 Introduction

Iron chalcogenides exhibit considerable promise for diverse applications within the realms of light energy conversion, hydrogen evolution, high-temperature superconductors, batteries, energy storage devices, and high-capacity capacitors [[1], [2], [3], [4], [5], [6], [7]]. Ongoing research in the exploration of iron chalcogenides, particularly in the context of supercapacitors and photovoltaic devices leveraging their magnetic attributes, has attracted substantial attention [[8], [9], [10], [11], [12]]. Among these materials, iron selenide stands out for its significant absorption coefficient, narrow band gap, and potential utility in photovoltaic cells [[10], [11], [12], [13], [14], [15]]. Consequently, iron selenide has emerged as a viable alternative to conventional silicon (Si) in photovoltaic applications [[16], [17], [18], [19]]. Noteworthy is the fact that iron selenides exhibit the dual properties of semiconductors and superconductors, coupled with magnetic light absorption capabilities [[20], [21], [22]]. The pyrite crystal structure, particularly due to its isotropic nature in all three dimensions, presents intriguing applications spanning geochemistry, catalysis, and photovoltaic energy conversion [[23], [24], [25], [26], [27], [28], [29]]. Various preparation techniques, including physical vapor deposition (PVD), thermal vapor oxidation/sulfurization [[30], [31], [32], [33]], electrochemical deposition [25], solvothermal reactions [34], vapor-liquid-solid mechanisms [35], and spray pyrolysis [[36], [37], [38]], have been employed to synthesize $FeX_{2}$ (X = Se, S) samples.

Moreover, the fabrication of thin films of $Fe{Se}_{2}$ and the enhancement of their crucial properties have garnered considerable interest. However, it is crucial to note that while $FeS_{2}$, $Fe{Se}_{2}$, and iron oxide represent novel materials with promising attributes, further refinement of their properties is imperative to enable effective utilization across various applications. Alloying strategies have emerged as a viable approach to augment these materials' properties, offering a potential avenue for improvement [Insert relevant citations as needed].Sun and Ceder's seminal study [39] investigated the possibility of engineering the band gap of Iron pyrite through the introduction of specific metallic dopants such as Zn, Os, Ru, Hg, Ba, and others. Employing first-principles calculations, their research aimed to ascertain the possibility of widening the band gap of Iron pyrite via doping, conclusively demonstrating the potential of numerous doping elements to expand the band gap of pyrite. Interestingly, certain compounds like ${ZnS}_{2}$, $RuS_{2}$, and $OsS_{2}$ exhibited larger band gaps than Iron pyrite, emphasizing the incorporation of group II and Cd within the pyrite structure. In a complementary investigation, Lehner et al. [40] illuminated the influence of doping pyrite crystals with Ni, As, or Co on their electronic characteristics. The doping of Iron pyrite has garnered attention from several researchers. Ferrer et al. [41] scrutinized electronic characteristics of FeS alloyed with (Al, Cu, Ni), revealing that the alloyed FeS samples exhibited n-type behavior. Xiao et al. [42] reported that Zn alloyed with $FeS_{2}$ films led to an elevation of the band gap, spanning from 0.95 to 1.14 eV. Furthermore, Sai et al. [32] demonstrated that Zn doping could elevate the band gap of ${Zn}_{x}{Fe}_{1-x}S_{2}$ samples, starting from 0.95 eV and reaching 1.16 eV for lower Zn concentrations. However, as Zn concentrations increased, the band gap gradually decreased to 0.70 eV. Their findings were validated through experimental studies and Density Functional Theory (DFT) calculations, with a noteworthy emphasis on the high optical absorption characteristics, affirming Iron pyrite as a material showing potential for use in solar energy applications.

Notably, improvement in the band gap was primarily achieved in Ru and Os -doped compositions; however, their integration into pyrite was constrained by crystallographic limitations. Furthermore, $FeS_{2}$ pyrite doping with oxygen was found to elevate the band gap according to DFT calculations [42]. Overall, ruthenium emerged as the highly favorable option for augmenting the band gap of Iron pyrite films while enhancing their optical and electrical characteristics.

The primary objective of the current study is to investigate the impact of alloying $Fe{Se}_{2}$ with ruthenium. This choice is informed by the substantial structural similarities between $Fe{Se}_{2}$ and $FeS_{2}$ layers, both of which are prepared using the same spray pyrolysis technique. Transition metal selenides have garnered significant interest in the research community due to their potential applications as efficient electrocatalysts for water splitting and as anodes for batteries [[43], [44], [45], [46], [47], [48], [49], [50]].

## 2 Experimental detailed

In this investigation, the spray pyrolysis method was utilized to synthesize thin films of Ru-doped iron oxide, incorporating varying molar ratios of ${RuCl}_{3}$. ${3H}_{2}O:{FeCl}_{3}\;.{6H}_{2}O$. Specifically, the explored molar ratios in this research covered values of x ≅ 0.011, 0.02, 0.15, and 0.20. The synthesis process commenced with the preparation of an aqueous solution of ${FeCl}_{3}\;.{6H}_{2}O$ at a concentration of 0.05 M. Subsequently, varying amounts of an aqueous solution of ${RuCl}_{3}$. ${3H}_{2}O$, adjusted to the specified molar ratios, were introduced to form the desired mixtures.

A primary challenge addressed in this study was the fabrication of thin films at a low temperature of 350 °C. To achieve this, all solutions were dispensed through a spray nozzle onto heated glass substrates, maintaining a nozzle-substrate distance of approximately 55 cm. Compressed air served as a carrier gas, with a jet rate of around 5 ml/min.

The synthesis process involved successive spraying of the aforementioned mixtures of aqueous solutions, namely ${RuCl}_{3}$. ${3H}_{2}O$ and ${FeCl}_{3}\;.{6H}_{2}O$ (both at a concentration of 0.05 M), each lasting 5 min. This spray deposition procedure was repeated for various concentrations, corresponding to the specified molar ratios of ${RuCl}_{3}$. ${3H}_{2}O$. Subsequently, selenium was introduced to the layers by placing selenium hemispheres within a test tube subjected to vacuum pressure $(∼10^{-4}\;Pa$). The resulting samples were then heated in an oven at 350 °C for 5 h.

**Fig. 1:**
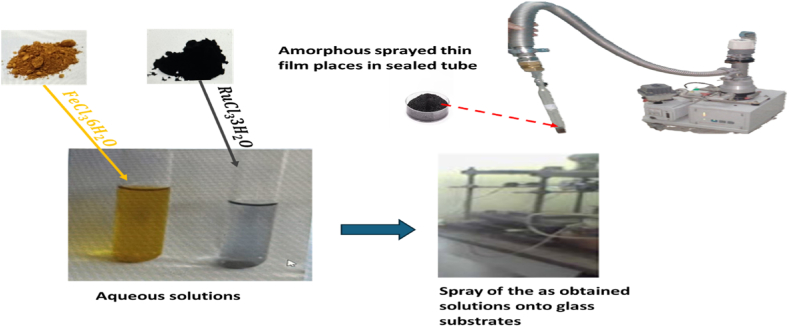
Steps of preparation.

The central focus of this study is to elucidate the influence of ruthenium concentration and temperature on the enhancement of the properties of synthesized thin films see Fig. 1.

## 3 Findings and discourse

### 3.1 XRD analysis

Subsequent to thermal processing at 350 °C, the synthesized phases underwent analysis through powder X-ray diffraction (PXRD) techniques, employing a Bruker D8 advanced diffractometer equipped with a lynxeye detector. The X-ray source employed was ${CuK}_{\alpha 1/\alpha 2}$, utilizing dichromatic copper radiation. The diffraction measurements were conducted in the θ-2θ geometry, scanning within the range of 2θ from 5 to 70°.

**Fig. 2:**
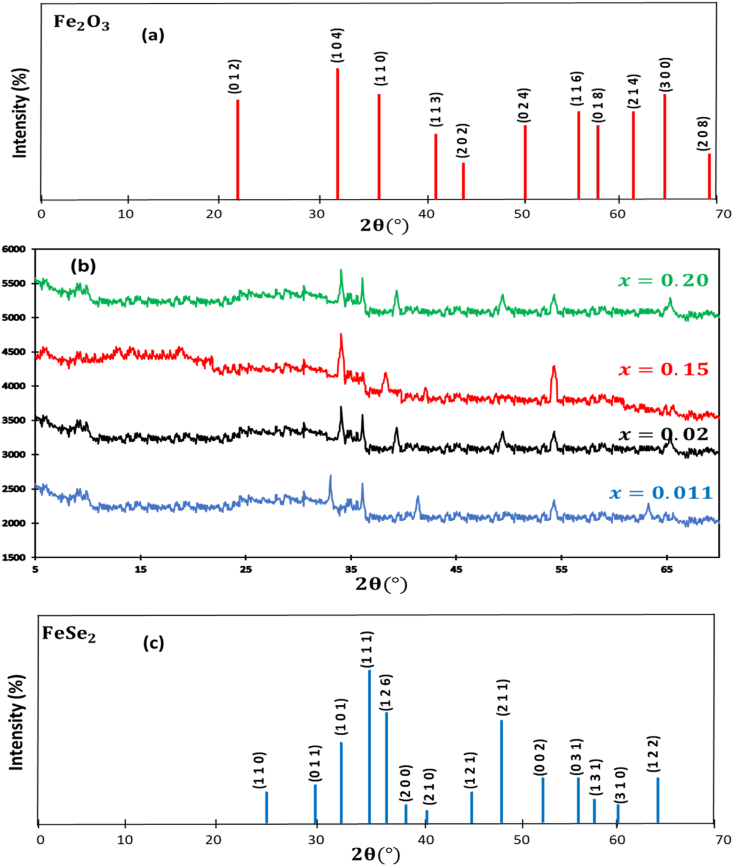
(a) XRD of ${\text{Fe}}_{2}O_{3}$; (b) XRD diffraction patterns of Ru-alloyed iron oxide films; (c) XRD of $\text{Fe}{\text{Se}}_{2}$.

To generate the diffraction patterns presented in this study, a comprehensive data acquisition approach was employed. Specifically, the measurement conditions involved utilizing the full diffraction power, maintaining Δθ at 0.017°, and employing a step size of 40 mA/40 KA, with a 3-s dwell time at each step. The identification of the phases in the samples was achieved by comparing the obtained diffraction data with the American Society for Testing and Materials (ASTM) phase database. This phase identification process was facilitated through the use of Powder Diffraction File (PDF) resources and further assisted by the application of Panalytical Xpert ProHigh Score software, ensuring accurate and reliable phase determination. Fig. 1 present XRD patterns. Following the heat treatment at 350 °C for 5 h in a sealed tube, under a vacuum of approximately $10^{-4}$ Pa, for the amorphous sprayed Ru-doped iron oxide films (with varying Ru concentrations of 1.1%, 2%, 15%, and 20%), the analysis revealed the exclusive presence of the ${\text{Fe}}_{2}O_{3}$ (Fig. 2. (a)) (JCPDS card n°: 01-002-0917) in the layers alloyed with 1.1% and 2% of ruthenium. This observation indicates that there was no selenization taking place in this procedure.

Furthermore, it is noteworthy that a significant proportion of the peaks observed in the XRD patterns (Fig. 2. (b)) can be attributed to the crystal alignments of $\text{Fe}{\text{Se}}_{2}$ (Fig. 2. (c)), specifically (111), (120), (211), and (101). Consequently, it is prudent to conclude that at the temperature for annealing of 350 °C, conducted in a selenium-rich environment with a partial pressure of $10^{-4}$ Pa for a duration of 5 h, the molar ratio of 2% represents an optimal level of alloying. This optimal ratio facilitates the preferential formation of the $Fe{Se}_{2}$ phase over iron oxide. This outcome is indicative of the favorable incorporation of selenium within the processed layers and the subsequent creation of the $Fe{Se}_{2}$ state at a reduced temperature, showcasing the advantageous kinetics associated with this process.

### 3.2 Analysis using Energy Dispersive X-ray spectroscopy (EDX or EDS)

To analyze the compositions of the Ru-alloyed $Fe{Se}_{2}$ stacked layers, Energy Dispersive X-ray (EDX) examinations were conducted utilizing a Thermo Scientific UltraDry Detector. The summarized analytical results are presented in Table 1. The outcomes reveal the consistent presence of selenium in the composition of the layers across various annealing temperatures, confirming the initiation of selenization at a relatively modest thermal level 350 °C. However, at this specific temperature during annealing, the selenium content proved insufficient to produce the $Fe{Se}_{2}$ phase, with only the iron oxide states reliably detected. This aligns with the results of the X-ray diffraction (XRD) analyses, as depicted in Fig. 1.

**Table 1:** EDS analysis of Ru-doped $\text{Fe}Se_{2}$ films synthesized via selenium atmosphere selenization.

Temperature	% of Atom Fe	% of Atom Se	% of Atom O	% of Atom Ru	(Ru/Fe)
350°C	25.6	11.48	46.51	0.287	= 0.0112
350°C	46.72	14.88	25.20	0.967	= 0.0207
350°C	25.78	14.96	48.1	3.874	= 0.1503
350°C	23.18	7.99	41.07	4.645	= 0.2004

Importantly, the layers consistently exhibited the existence of four elemental constituents: Fe, O, Se, and Ru, across different annealing temperatures and ruthenium concentrations. This observation strengthens the validity of the recognized stages outlined in the XRD patterns, specifically the Ru-doped ${Fe}_{2}O_{3}$ , Ru-alloyed ${Fe}_{3}O_{4}$, and Ru-alloyed $Fe{Se}_{2}$ phases.

### 3.3 Optical properties

The objective of the ensuing analyses was to investigate the influence of varied ruthenium concentrations and annealing temperatures on the optical properties, specifically the band gap values, of the Ru-alloyed $Fe{Se}_{2}$ films synthesized in this study. To achieve this, we subjected the amorphous Ru-alloyed iron oxide films to distinct heat treatment temperature 350 °C for a duration of 5 h within a Rapid Thermal Processing (RTP) oven. These treatments corresponded to different molar ratios of [RuCl3.3H2O][FeCl3.6H2O] be equal (0.011, 0.02, 0.15, and 0.20.).

Subsequently, the reflectance and transmittance of the resultant samples were measured using a SHIMADZU 3100S spectrophotometer. We used Eq (1) to determine the absorption coefficient, α [53]:

Eq (1) $$\alpha =\frac{1}{e}\ln\left[\frac{{\left(1-R\right)}^{2}}{2T}+{\left[\frac{\left(1-R\right)}{4T^{2}}+R^{2}\right]}^{\frac{1}{2}}\right]$$

here, T and R represent the transmittance and reflectance of the processed coating, respectively, while 'e' signifies the thickness of the layer. The thickness 'e' was determined using profilometry techniques and measured to be approximately 1.2 × $10^{-6}$ m, equating to 1.2 μm.

Through these measurements and calculations, we obtained the energies associated with various permitted transitions within the material, enabling us to characterize its optical properties.

**Fig. 3:**
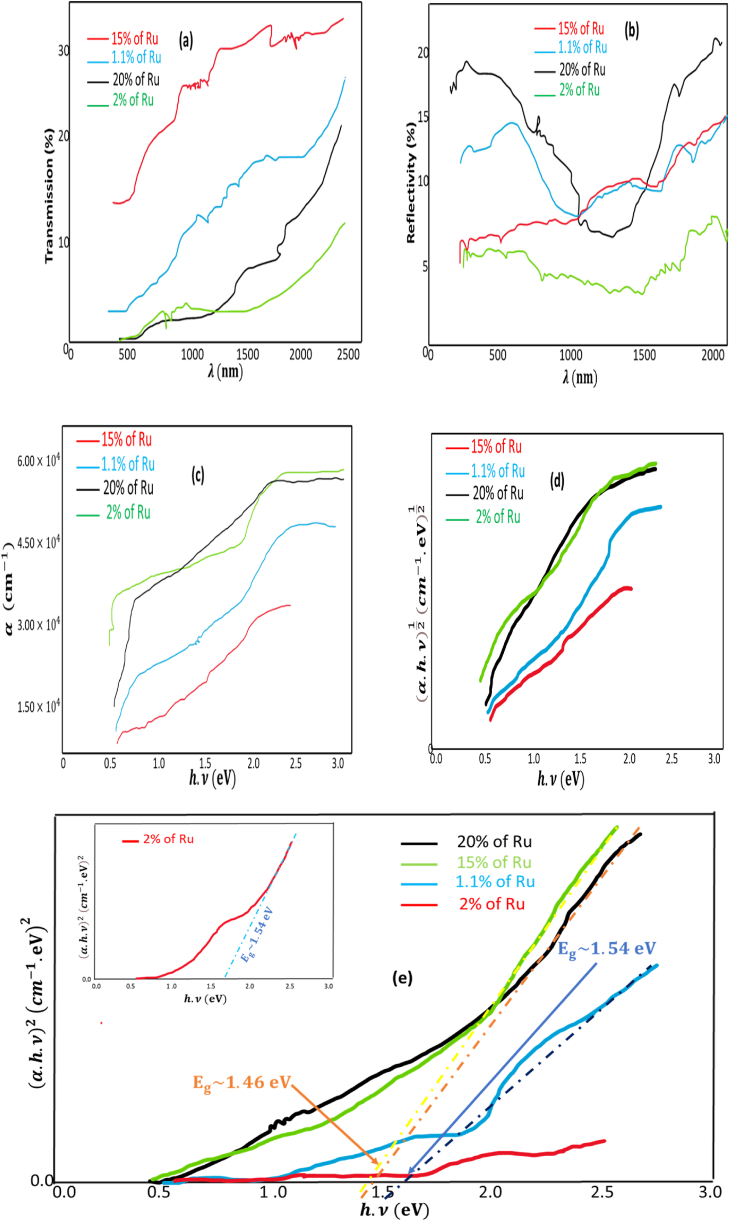
Characteristics related to the optics of Ru-doped $\text{FeS}e_{2}$ samples with varying concentrations of ruthenium (1.1%, 2%, 15%, and 20%), $\left(\left(a\right)\;T\left(\lambda \right),\left(b\right)\;R\left(\lambda \right),\left(c\right)\;\alpha \left(h\nu \right),\left(d\right){\left(\alpha h\nu \right)}^{\frac{1}{2}}\;\left(h\nu \right),\text{and}\;\left(e\right){\left(\alpha h\nu \right)}^{2}\;\left(h\nu \right)\right)<!--Q3:\text{Figures}\;\text{were}\;\text{not}\;\text{sequentially}\;\text{cited}\;\text{in}\;\text{the}\;\text{text},\text{and}\;\text{have}\;\text{been}\;\text{renumbered}\;\text{both}\;\text{in}\;\text{the}\;\text{text}\;\text{and}\;\text{in}\;\text{the}\;\text{artwork}.\;\text{Please}\;\text{check},\text{and}\;\text{correct}\;\text{if}\;\text{necessary}.-->\text{.}$.

The optical characteristics of the specimens, which underwent annealing at 350 °C in a selenium atmosphere at a vacuum pressure of approximately ∼ $10^{-4}$ Pa, are illustrated in Fig. 3. The results pertain to various ratios based on moles of [RuCl3.3H2O][FeCl3.6H2O] about (0.011, 0.02, 0.15, and 0.20.).

Fig. 3(a) and (b) reveal that the transmittance remains below 50% for wavelengths less than 1000 nm, while the reflectance stays low, below 20%, for wavelengths below 1000 nm. This data confirms the effective absorption characteristics of the treated films. Furthermore, the plotted curves, representing the absorption coefficient as a function of photon energy, unequivocally establish the substantial absorbance exhibited by the resultant layers (α > $10^{4}{cm}^{-1}$ for λ < 1000 nm). With the dominant phase being unequivocally identified as $Fe{Se}_{2}$ at the specified annealing temperature, as depicted in Fig. 1, particularly in the context of the molar ratio of 0.0112, it is plausible to infer that the introduction of Ru into the composition of the FeSe2 films has yielded enhancements in their optical characteristics. Therefore, it is admissible to assert that, at the specified temperature for alloy formation of 350 °C, the resultant Ru-doped $Fe{Se}_{2}$ films exhibit intriguing optical properties.

By plotting ${\left(\alpha hv\right)}^{\frac{1}{2}}$ and ${\left(\alpha hv\right)}^{2}$ against the photon energy (hv) various permitted transition energy levels, including 2.01 eV, 2.1 eV, and 2.07 eV, were derived. No discernible energy value associated with an indirect transition was discerned. Notably, only the graphical representation of the ${\;\left(\alpha hv\right)}^{2}$ versus hv relationship, as illustrated in Fig. 3(e), manifests a linear correlation, indicating unequivocally that the Ru-doped $Fe{Se}_{2}$ samples exhibit a direct band gap energy across the spectrum of proportions based on moles under consideration as seeing in Fig. 3(d). Hence, it can be surmised that the Ru-alloyed $Fe{Se}_{2}$ pyrite layers in question have retained a direct transition consistent with their band gap properties at the specified annealing temperature of 350 °C. Such a phenomenon holds significant promise for their potential application in the realm of multispectral photovoltaic cells, bearing considerable scientific and practical interest.

Following the incorporation of ruthenium into the composition of $Fe{Se}_{2}$ layers, several discernible changes have transpired. Notably, there has been an observable shift in the two-theta values towards higher angles, accompanied by broadening in the X-ray diffraction (XRD) peaks, as depicted in Fig. 1. Furthermore, a conspicuous increase in the energy band gap has been observed, escalating from 1.03 eV to 1.54 eV, a trend particularly pronounced for the molar ratio of 0.02 and 0.0112, as illustrated in Fig. 3. These empirical findings strongly indicate the successful substitution of Ru ions into the lattice structure of $Fe{Se}_{2}$, thus providing a rationale for the emergence of new minor phases that can be attributed to the ${Ru}_{x}{Fe}_{\left(1-x\right)}{Se}_{2}$ compounds.

The pertinent related direct band gap energies to various Ru-doped phases, achieved through the incorporation of distinct proportions based on moles, have been comprehensively tabulated in Table 2. A meticulous examination of Table 2 reveals that the band gap energies associated with the diverse Ru-doped $Fe{Se}_{2}$ phases fall within the range of 1.46–1.54 (eV). These values are notably well-suited for a multitude of applications, with particular relevance to the domain of photovoltaics.

**Table 2:** Band gap analysis of Ru-doped $\text{Fe}Se_{2}$ films via selenium annealing in RTP oven.

Molar ratios $x=\frac{\left[Ru\right]}{\left[Fe\right]}$	Corresponding ${Ru}_{x}{Fe}_{\left(1-x\right)}{Se}_{2}$	Band gap (eV) (direct transition)
0.0112	${Ru}_{0.011}{Fe}_{0.989}{Se}_{2}$	1.54 (eV)
0.02	${Ru}_{0.02}{Fe}_{0.98}{Se}_{2}$	1.54 (eV)
0.15	${Ru}_{0.15}{Fe}_{0.85}{Se}_{2}$	1.46 (eV)
0.20	${Ru}_{0.2}{Fe}_{0.8}{Se}_{2}$	1.46 (eV)

Of particular interest are the band gap energies corresponding to molar ratios of 0.0112 and 0.02, which approximate 1.54 eV—a proximity to the coveted 1.5 eV threshold for solar energy applications involving thin semiconductor films. However, it is imperative to note that the layers doped with a proportion based on moles of 0.02 exhibit higher absorbance compared to those doped with a proportion based on moles of 0.0112.

From these findings, one may deduce that the introduction of ruthenium into the composition of $Fe{Se}_{2}$ layers, especially at the proportions based on moles of 0.02, promotes the emergence of pyrite FeSe2 phases within the material.

### 3.4 Surface structure analysis: scanning electron microscopy (SEM)

**Fig. 4:**
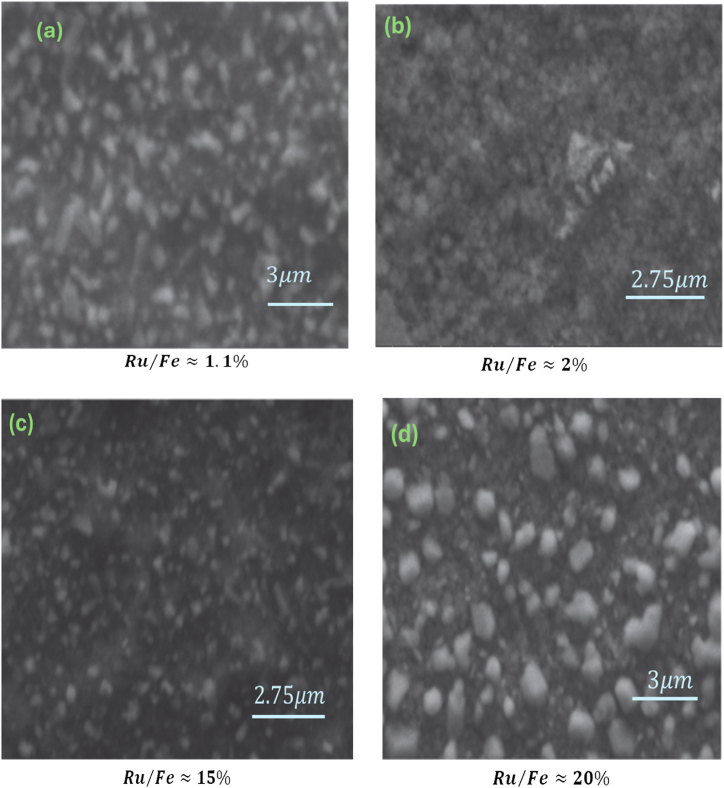
SEM surface.

The surface structures of the Ru-doped $Fe{Se}_{2}$ films were systematically characterized using a Thermo Scientific Q250 Scanning Electron Microscope (SEM). Illustrated in Fig. 4 (a, b, c and d) are SEM micrographs providing detailed views of the film surfaces, following a meticulous heat treatment process conducted under a selenium atmosphere at a vacuum pressure of approximately ∼ $10^{-4}$ torr for a duration of 5 h. These films, originating from spray-deposited Ru-alloyed iron oxide precursors, underwent annealing at temperature 350 °C.

The SEM images reveal a porous surface structure characterized by an inhomogeneous granular arrangement. This porous and inhomogeneous structure possesses significant relevance for potential applications, such as serving as materials for battery anodes and acting as effective electrocatalysts for complete water splitting [[43], [44], [45], [46], [47], [48], [49], [50], [51], [52]]. The observed porous surface structure aligns coherently with the substantial absorbance noted in Fig. 3(c).

It is noteworthy that alterations in the surface morphology were discerned in relation to both ruthenium concentration and annealing temperature. These variations can be ascribed to the deliberate integration of ruthenium into the layer's composition. Significantly, the grains within the inhomogeneous structure appeared to enlarge, validating the observed enhancement in absorbance characteristics for the corresponding layers. This underscores the influence of ruthenium not only on the composition but also on the morphology of the obtained films.

### 3.5 Electrical properties

The integration of ruthenium into the composition of $Fe{Se}_{2}$ layers was executed using identical proportions as previously established. Consequently, this Ru-substitution process has engendered novel phases denoted as ${Ru}_{x}{Fe}_{\left(1-x\right)}{Se}_{2}$, exhibiting distinct and superior properties compared to their non-alloyed $Fe{Se}_{2}$ counterparts, thereby rendering them viable for diverse applications.

**Fig. 5:**
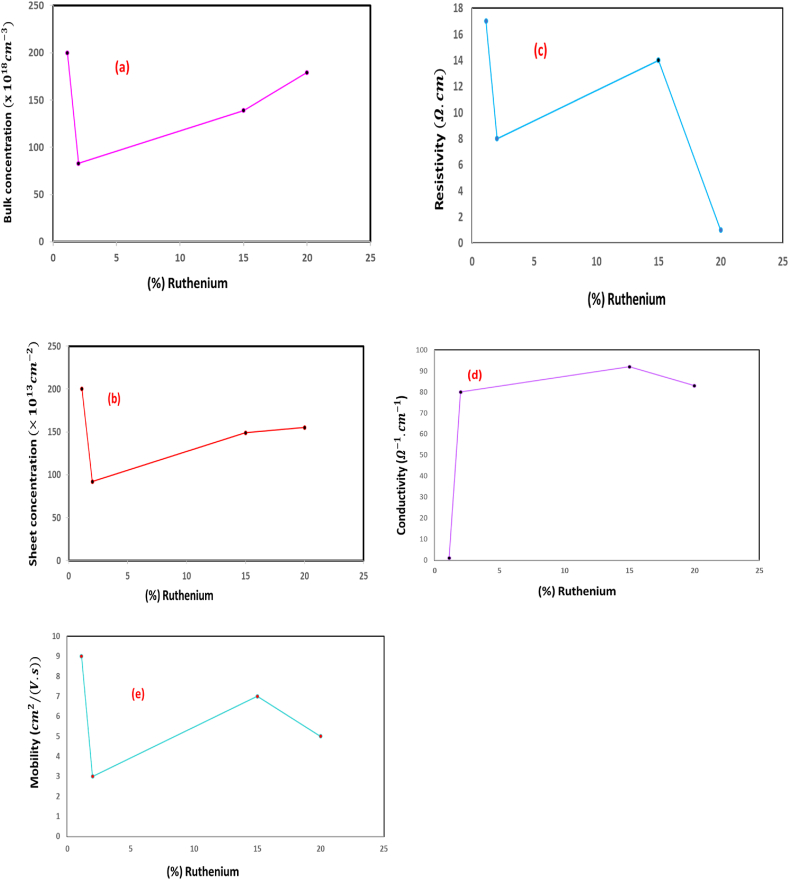
Dependence of the bulk composition(a), sheet composition (b), resistivity (c), conductivity (d), and mobility (e) of the Ru-alloyed $FeSe_{2}$ films.

In-depth analyses of the electrical characteristics of the Ru-doped $Fe{Se}_{2}$ layers have been conducted. These investigations encompassed an examination of conductivity type, mobility, sheet carrier concentration, bulk concentration, resistivity, and conductivity. These studies were performed on the resultant layers subsequent to the introduction of varying quantities of ruthenium, as per the prescribed ratios (0.15, and 0.20), followed by annealing at 350 °C, as depicted in Fig. 5.

The electric characteristics of the layers combined with various proportions based on moles and subsequently subjected to annealing processes at temperature of 350 °C were meticulously characterized through measurements. These measurements were conducted subsequent to the use of a magnetic field with an intensity denoted as B = 0.554 T.

It is noteworthy that all layers subjected to investigation exhibited N-type conductivity, as is indicated by the collected data. Fig. 5 serves as a graphical representation, visually encapsulating the findings garnered during this comprehensive study pertaining to the electric characteristics of Ru-doped $Fe{Se}_{2}$ layers.

Fig. 5(a) displays the graphical representations of the bulk concentration dependencies in relation with respect to the doping proportions based on moles and a thermal treatment temperature. In Fig. 5(b), we present the observed influence of both the proportions based on moles and a thermal treatment temperature on the sheet composition of the obtained layers. To comprehensively illustrate the effects of alloying molar ratio and annealing temperature on the electrical properties, Fig. 5(c) and (d) depict the resistivity and conductivity, respectively, as functions of these variables.

Moreover, our investigation extends to the analysis of layer mobility in response to changes in alloying molar ratio and annealing temperature. This examination culminates in the presentation of mobility-dependent curves, as portrayed in Fig. 5(e).

A discernible trend emerges as the bulk concentration exhibits a pronounced increase with the elevation of annealing temperature, as elucidated in Fig. 5(a)). Of note, the highest bulk concentration, slightly below $2\times 10^{20}{cm}^{-3}$, was discerned in the sample annealed at 350 °C with a 1,1% ruthenium alloying ratio. Conversely, when employing a 20% alloying molar ratio, the bulk concentration exhibited an even higher value (∼1.79 × 1 $10^{20}{cm}^{-3}$) in layers annealed at 350 °C.

Similar to the bulk concentration, the sheet concentration showed a parallel increase in response in annealing temperature and alloying molar ratio, as depicted in Fig. 5(b). The zenith sheet concentration, slightly below $2\times 10^{15}{cm}^{-2}$, was noted for the sample doped with 1.1% ruthenium. With a 20% doping ratio, the sheet composition reached approximately $1.55\times 10^{15}{cm}^{-2}$ for layers annealed at 350 °C.

The crux of our findings lies in the conductivity measurements, with layers subjected to annealing at 350 °C and doped at molar ratios of 0.02, 0.15, and 0.20 exhibiting the most pivotal results, as exemplified in Fig. 5(d). Moreover, samples annealed at 350 °C displayed lower conductivity, resulting in higher resistivity values, as presented in Fig. 5(c).

Furthermore, the mobility of the layers was scrutinized concerning changes in the thermal treatment temperature and alloying ratios, and the results are illustrated in Fig. 5(e). The maximum mobility value (∼9 ${cm}^{2}/\left(V.s\right))$ was ascertained for samples doped with 1.1% ruthenium and thermal treatment temperature at 350 °C. This value reduced in cases of doping ratios of 0.02, 0.15 and 0.20, only to subsequently rise and reach its zenith for a 0.20 doping ratio. Mobility exhibited a increasing trend with an increase in the molar ratio, with an augmentation observed when the doping proportions based on moles reached 0.15. Moreover, by transitioning the alloying proportions based on moles from 0.02 to 0.15, enhanced mobility values before subsequently diminishing.

Furthermore, the refinement of the $Fe{Se}_{2}$ amendment following alloying with ruthenium necessitates a more advanced structural characterization, as demonstrated by Shoushuang Huang et al. [51]. It is noteworthy that adept control over nano/microscale structures, particularly in the case of ruthenium substitution, can significantly influence the precise modulation of ruthenium content and enhance the durability of the resultant layers.

## 4 Conclusion

Ru-doped $Fe{Se}_{2}$ samples were synthesized employing a straightforward methodology, commencing with the deposition of an aqueous solution containing RuCl3·3H2O at varied concentrations (0.0112, 0.02, 0.15, and 0.20) onto a pre-sprayed solution of water of FeCl3·6H2O. The resultant films exhibited amorphous characteristics and were subsequently subjected to heat treatment under a selenium-rich atmosphere at low temperature 350 °C. Notably, selenium incorporation into the Ru-doped iron oxide films commenced at a relatively low thermal level of 350 °C, marking a significant achievement.

Upon thermal treatment, these Ru-alloyed $Fe{Se}_{2}$ layers exhibited the emergence of minor phases, substantiating the successful integration inclusion of ruthenium within their structure. This can be ascribed to the presence of ${Ru}_{x}{Fe}_{\left(1-x\right)}{Se}_{2}$ phases. The introduction of ruthenium into the $Fe{Se}_{2}$ layers resulted in observable changes, such as a two-theta shift and broadening of X-ray diffraction peaks in the X-ray Diffraction (XRD) to elevated levels and an increase in the energy band gap from 1.03 to 1.54 eV. Remarkably, at an annealing thermal level of 350 °C and an doping ratio of 1.1% and 2%ruthenium, only the singular state of $Fe{Se}_{2}$ was acquired, with the band gap energy in alignment with the Ru-doped $Fe{Se}_{2}$ phase measuring 1.50 eV, indicative of a direct transition. This phenomenon encourages the exploration of these films for use in multispectral photovoltaic applications.

Notably, following the introduction of ruthenium, all the resulting films exhibited N-type conductivity. These results underscore the judicious selection of ruthenium as an alloying material for enhancing the properties of $Fe{Se}_{2}$-pyrite thin films. Furthermore, the structural modifications of the $Fe{Se}_{2}$ structure upon Ru-substitution, open avenues for further development. This underscores the capacity to precisely tailor the quantity of added ruthenium and the endurance of the resultant layers, particularly at the nano/microscale level.

The amelioration of $Fe{Se}_{2}$ layers in terms of their structural, optical, and electrical properties has the potential for diverse applications, including serving as cost-effective materials for solar photovoltaic cell, materials used as anodes in batteries, and effective electrocatalysts for processes for comprehensive water splitting and the generation of hydrogen through photocatalysis.

The scope of this study is constrained by its exclusive reliance on experimental results. Future endeavors could expand the research framework to encompass theoretical analyses, akin to the investigations conducted by Sai in the context of ruthenium alloyed iron pyrite [[32], [33], [34], [35], [36], [37], [38], [39], [40], [41], [42], [43], [44], [45], [46], [47], [48], [49], [50], [51], [52]]. Additionally, there is potential for investigating the influence of zinc alloying on the properties of $Fe{Se}_{2}$. This approach would contribute to a more comprehensive understanding of the material's characteristics and behavior.

## Data availability statement

Data will be made available on request.

## Additional information

No additional information is available for this paper.

## CRediT authorship contribution statement

**Eman A. Alghamdi:** Writing – original draft. **Refka Sai:** Writing – original draft.

## Declaration of competing interest

The authors declare that they have no known competing financial interests or personal relationships that could have appeared to influence the work reported in this paper.

## References

[1] Macpherson H.A., Stoldt C.R. (2012). Iron pyrite nanocubes: size and shape considerations for photovoltaic application. ACS Nano.

[2] Jasion D., Barforoush J.M., Qiao Q., Zhu Y., Ren S., Leonard K.C. (2015). Low-dimensional hyperthin FeS2 nanostructures for efficient and stable hydrogen evolution electrocatalysis. ACS Catal..

[3] Yin Z., Haule K., Kotliar G. (2011). Kinetic frustration and the nature of the magnetic and paramagnetic states in iron pnictides and iron chalcogenides. Nat. Mater..

[4] Wang Shixuan, Zhao Xiangyu (2023). Minireview on metal–Chalcogenide Cathode materials with dual-Redox Centers for Magnesium-metal-anode-based batteries: recent progress and future directions. Energy Fuels.

[5] Wang Y.-X., Yang J., Chou S.-L., Liu H.K., Zhang W.-x., Zhao D., Dou S.X. (2015). Uniform yolk-shell iron sulfide–carbon nanospheres for superior sodium–iron sulfide batteries. Nat. Commun..

[6] Barawi M., Ferrer I.J., Flores E., Yoda S., Ares J.R., anchez C.S. (2016). Hydrogen photoassisted generation by visible light and an earth abundant photocatalyst: pyrite (FeS2). J. Phys. Chem. C.

[7] Wang Y.-X., Yang J., Chou S.-L., Liu H.K., Zhang W.-x., Zhao D., Dou S.X. (2015). Uniform yolk-shell iron sulfide–carbon nanospheres for superior sodium–iron sulfide batteries. Nat. Commun..

[8] Akhtar M., Akhtar J., Malik M.A., Tuna F., Helliwell M., O'Brien P. (2012). Deposition of iron selenide nanocrystals and thin films from tris (N, N-diethyl-N0- naphthoylselenoureato) iron (iii). J. Mater. Chem..

[9] Akhtar M., Abdelhady A.L., Malik M.A., O'Brien P. (2012). Deposition of iron sulfide thin films by AACVD from single source precursors. J. Cryst. Growth.

[10] Akhtar M., Malik M.A., Raftery J., O'Brien P. (2014). Synthesis of iron selenide nanocrystals and thin films from bis (tetraisopropyldiselenoimidodiphosphinato) iron (II) and bis (tetraphenyldiselenoimidodiphosphinato) iron (II) complexes. J. Mater. Chem..

[11] Akhtar M., Akhter J., Malik M.A., O'Brien P., Tuna F., Raftery J., Helliwell M. (2011). Deposition of iron sulfide nanocrystals from single source precursors. J. Mater. Chem..

[12] Akhtar M., Malik M.A., Tuna F., O'Brien P. (2013). The synthesis of iron sulfide nanocrystals from tris (O-alkylxanthato) iron (III) complexes. J. Mater. Chem..

[13] Piperno L., Celentano G., Sotgiu G. (2023). Electrodeposition of iron selenide: a review. Coatings.

[14] Xu J., Jang K., Lee J., Kim H.J., Jeong J., Park J.-G., Son S.U. (2011). Phase-selective growth of assembled FeSe2 nanorods from organometallic polymers and their surface magnetism. Cryst. Growth Des..

[15] Xu Siyuan, Yin Yiheng, Niu Huan, Wang Xiting, Shao Chen, Xi Kai, Zhang Zhaofu, Guo Yuzheng (2021). Adsorption and diffusion of alkali atoms on FeX2 (X = Se, S) surfaces for potassium-ion battery applications. Appl. Surf. Sci..

[16] Yuan B.X., Luan W.L., Tu S.T. (2012). One-step synthesis of cubic FeS2 and flower-like FeSe2 particles by a solvothermal reduction process. Dalton Trans..

[17] Xu J., Jang K., Lee J., Kim H.J., Jeong J., Park J.G., Son S.U. (2011). Phase-Selective growth of assembled FeSe2 nanorods from organometallic polymers and their surface magnetism. Cryst. Growth Des..

[18] Hou B., Benito-Alifonso D., Webster R.F., Cherns D., Galan M.C., Fermín D.J. (2021). Synthetic mechanism studies of iron selenides: an emerging class of materials for electrocatalysis. Catalysts.

[19] Oyetunde Temidayo, Omorogie Martins O., O'Brien Paul (2020). Ferromagnetic FeSe2 from a mixed sulphur-selenium complex of iron [Fe{(SePPh2NPPh2S)2N}3] through pyrolysis. Heliyon.

[20] Ghalawat, M; Poddar, P, Remarkable effect of Fe and Se composition on magnetic properties-comparative study of the Fe-Se system at the nanoscale, J. Phys. Chem. C, Volume126 Issue 9, Page 4655-4663..

[21] Ovchenkov Y.A., Chareev D.A., Kulbachinskii V.A., Kytin V.G., Presnov D.E., Skourski Y., Volkova O.S., Vasiliev A.N. (2018). Magnetotransport properties of FeSe in fields up to 50 T. J. Magn. Magn Mater..

[22] Hsu F.C., Luo J.Y., Yeh K.W., Chen T.K., Huang T.W., Wu P.M., Lee Y.C., Huang Y.L., Chu Y.Y., Yan D.C., Wu M.K. (2008). Superconductivity in the PbO-type structure α-FeSe. Proc. Natl. Acad. Sci. U.S.A..

[23] Anantharaj Sengeni, Noda Suguru (2020). Nickel selenides as pre-catalysts for electro- chemical oxygen evolution reaction. Int. J. Hydrogen Energy.

[24] Azadar Hussain Raja, Hussain Iqtadar (2020). Copper selenide thin films from growth to applications. Solid State Sci..

[25] Pesko Edyta, Zero Zukowska Elzbieta, Krzton-Maziopa Anna (2020). Electrocrystallization of nanostructured iron-selenide films for potential appli- cation in dye sensitized solar cells. Thin Solid Films.

[26] Luo Minghe, Yu Haoxiang, Hu Feiyang, Liu Tingting, Shu Jie (2020). Metal selenides for high performance sodium ion batteries. Chem. Eng. J..

[27] Ao Kin Long, Shao Yangfan, Chan Iat Neng, Shi Xingqiang, Pan Hui (2020). Design of novel pentagonal 2D transitional-metal sulphide monolayers for hydrogen evolution reaction. Int. J. Hydrogen Energy.

[28] Jayaraman Theerthagiri Raja, Senthil Arumugam, Nithyadharseni Palaniyandy, Lee Seung Jun, Durai Govindarajan, Kuppusami Parasuraman, Madhavan Jagannathan, Choi Myong Yong (2020). Recent progress and emerging challenges of transition metal sulfides based composite electrodes for electro- chemical supercapacitive energy storage. Ceram. Int..

[29] Sahoo Surjit, Kumar Naik Kusha, Dattatray J. (2017). Late, Chandra Sekhar Rout, Electrochemical synthesis of a ternary transition metal sulfide nanosheets on nickel foam and energy storage application. J. Alloys Compd..

[30] Liu Hongfei, Chi Dongzhi (2016). Synthesis of iron sulfide and iron oxide nanocrystal thin films for green energy applications. Procedia Eng..

[31] Sai R., Gorochov O., Alghamdi E.A., Ezzaouia H. (2022).

[32] Sai R., Shawish I., Nofal M.M., Alghamdi E.A. (2023).

[33] Sai R., Gorochov O., Ezzaouia H. (2021).

[34] Luo Lingli, Luan Weiling, Yuan Binxia, Zhang Chengxi, Jin Lin (2015). High efficient and stable solid solar cell: based on FeS2 nanocrystals and P3HT: PCBM. Energy Proc..

[35] Srivastava Ravi P., Ingole Sarang (2022). Reduction in Urbach energy and density of states for pyrite (FeS2) thin films: healing of sulfur vacancies during hematite to pyrite transformation. J. Phys. Chem. Solid..

[36] Sai R., Abumousa R.A. (2023). Impact of iron pyrite nanoparticles sizes in photovoltaic performance. Coatings.

[37] Sai R., Ezzaouia H., Muaffaq M.M. (2021). Electronic structure of iron pyrite by the LMTO_ASA method. Results Phys..

[38] Nader Ghobadi, Sohrabi Parisa, Hatami Hamid Reza (2020). Correlation between the photocatalytic activity of CdSe nanostructured thin films with optical band gap and Urbach energy. Chem. Phys..

[39] Sun Ruoshi, Ceder Gerbrand (2011). Feasibility of band gap engineering of pyrite FeS2. Phys. Rev. B.

[40] Babedi L., Tadie M., von der Heyden B.P., Chareev D.A. (2023). A rest potential study of impurity (As, Au, Ni and Co) bearing synthetic pyrite in alkaline flotation conditions. Miner. Eng..

[41] Tripathi J., Chandrawat G.S., Singh J., Tripathi S., Sharma A. (2021). Correlation among local structure, magnetic, structural, and electronic properties in polyol synthesized iron sulfide (FeS2) nanoparticles. J. Alloys Compd..

[42] Xiao Pin, Fan Xiao-Li, Han Zhanga, Fang Xiaoliang, Liu Li-Min (2015). Increasing the band gap of FeS2 by alloying with Zn and applying biaxial strain: a first-principles study. J. Alloys Compd..

[43] Kong Fanjun, Zheng Jihui, Shi Tao, Qian Bin (2021). Electrochemical and electrocatalytic performance of FeSe2 nanoparticles improved by selenium matrix. Mater. Lett..

[44] Liu Yanzhen, Yang Chenghao, Li Youpeng, Zheng Fenghua, Li Yijuan, Deng Qiang, Zhong Wentao, Wang Gang, Liu Tiezhong (2020). FeSe2/nitrogen-doped carbon as anode material for potassium-ion batteries. Chem. Eng. J..

[45] Dong Shijia, Su Qingmei, Jiao Weicheng, Ding Shukai, Zhang Miao, Du Gaohui, Xu Bingshe (2020). FeSe2 microspheres coated with carbon layers as anode materials for sodium-ion batteries. J. Alloys Compd..

[46] Shwetharani R., Nagaraju D.H., Geetha Balakrishna R., Suvina V. (2019). Hydrogenase enzyme like nanocatalysts FeS2 and FeSe2 for molecular hydrogen evolution reaction. Mater. Lett..

[47] Fan Haosen, Hong Yu, Zhang Yufei, Guo Jing, Wang Zhen, Wang Hao, Zhao Ning, Zheng Yun, Du Chengfeng, Dai Zhengfei, Yan Qingyu, Xu Jian (2018). 1D to 3D hierarchical iron selenide hollow nanocubes assembled from FeSe2@C core-shell nanorods for advanced sodium ion batteries. Energy Storage Mater..

[48] Men Shuang, Lin Jiajv, Zhou Yuan, Kang Xiongwu (2021). N-doped porous carbon wrapped FeSe2 nanoframework prepared by spray drying: a potential large- scale production technique for high-performance anode materials of sodium ion batteries. J. Power Sources.

[49] Jujun Yuan, Wen Liu, Xianke Zhang, Yaohui Zhang, Wentao Yang, Weidong Lai, Xiaokang Li, Jiujun Zhang, Xifei Li, MOF Derived ZnSe–FeSe2/RGO..

[50] Jia Jia, Sun Wenjuan, Zhang Qiqi, Zhang Xiaozhuo, Hu Xiaoyun, Liu Enzhou, Fan Jun (2020). Inter-plane heterojunctions within 2D/2D FeSe2/g-C3N4 nanosheet semiconductors for photocatalytic hydrogen generation. Appl. Catal. B Environ..

[51] Huang Shoushuang, He Qingquan, Chen Wenlong, Zai Jiantao, Qiao Qiquan, Qian Xuefeng (2015). 3D hierarchical FeSe2 microspheres: controlled synthesis and applications in dye-sensitized solar cells. Nano Energy.

[52] Alghamdi E.A., Sai R. (OCTOBER 2023). Impact of alloying iron pyrite by ruthenium on its band gap values and its insight to photovoltaic performance. Heliyon.

[53] Carlos Martinez Anton Juan, Gomez Manzanares Angela, Alvarez Fernandez-Balbuena Antonio, Vazquez Molini Daniel (2021). Measuring the absorption coefficient of optical materials with arbitrary shape or distribution within an integrating sphere. Opt Express.

